# Macrophage membrane-camouflaged pH-sensitive nanoparticles for targeted therapy of oral squamous cell carcinoma

**DOI:** 10.1186/s12951-024-02433-4

**Published:** 2024-04-12

**Authors:** Lin Yang, Hongjiao Li, Aihua Luo, Yao Zhang, Hong Chen, Li Zhu, Deqin Yang

**Affiliations:** 1 Department of Endodontics, Stomatological Hospital of Chongqing Medical University, Chongqing, 404100 China; 2 Chongqing Key Laboratory of Oral Diseases and Biomedical Sciences, Stomatological Hospital of Chongqing Medical University, Chongqing, 404100 China; 3 Chongqing Municipal Key Laboratory of Oral Biomedical Engineering of Higher Education, Chongqing, 404100 China; 4 Chongqing Key Laboratory of Oral Diseases and Biomedical Sciences, 426 Songshi North Road, Yubei District, Chongqing, 401147 China; 5 Key Laboratory for Biorheological Science and Technology of Ministry of Education, State and Local Joint Engineering Laboratory for Vascular Implants, Bioengineering College of Chongqing University, Chongqing, 400044 China

**Keywords:** Oral squamous cell carcinoma, pH-sensitive, Macrophage membrane, Target delivery

## Abstract

**Background:**

Oral cancer is the most common malignant tumor of the head and neck, and 90% of cases are oral squamous cell carcinoma (OSCC). Chemotherapy is an important component of comprehensive treatment for OSCC. However, the clinical treatment effect of chemotherapy drugs, such as doxorubicin (DOX), is limited due to the lack of tumor targeting and rapid clearance by the immune system. Thus, based on the tumor-targeting and immune evasion abilities of macrophages, macrophage membrane-encapsulated poly(methyl vinyl ether *alt* maleic anhydride)-phenylboronic acid-doxorubicin nanoparticles (MM@PMVEMA-PBA-DOX NPs), briefly as MM@DOX NPs, were designed to target OSCC. The boronate ester bonds between PBA and DOX responded to the low pH value in the tumor microenvironment, selectively releasing the loaded DOX.

**Results:**

The results showed that MM@DOX NPs exhibited uniform particle size and typical core-shell structure. As the pH decreased from 7.4 to 5.5, drug release increased from 14 to 21%. The in vitro targeting ability, immune evasion ability, and cytotoxicity of MM@DOX NPs were verified in HN6 and SCC15 cell lines. Compared to free DOX, flow cytometry and fluorescence images demonstrated higher uptake of MM@DOX NPs by tumor cells and lower uptake by macrophages. Cell toxicity and live/dead staining experiments showed that MM@DOX NPs exhibited stronger in vitro antitumor effects than free DOX. The targeting and therapeutic effects were further confirmed in vivo. Based on in vivo biodistribution of the nanoparticles, the accumulation of MM@DOX NPs at the tumor site was increased. The pharmacokinetic results demonstrated a longer half-life of 9.26 h for MM@DOX NPs compared to 1.94 h for free DOX. Moreover, MM@DOX NPs exhibited stronger tumor suppression effects in HN6 tumor-bearing mice and good biocompatibility.

**Conclusions:**

Therefore, MM@DOX NPs is a safe and efficient therapeutic platform for OSCC.

**Supplementary Information:**

The online version contains supplementary material available at 10.1186/s12951-024-02433-4.

## Introduction

Oral cancer is among the most common malignancies of the head and neck, with an estimated 650,000 new cases reported annually worldwide [1]. Approximately 90% of oral cancers are oral squamous cell carcinoma (OSCC). Its pathogenesis has not been fully elucidated. The highest 5-year survival rate of patients with oral cancer is only 56–68% on average [2, 3]. At present, surgery, radiotherapy and chemotherapy are the main treatments for OSCC. Chemotherapy is an important component of the comprehensive treatment of OSCC. Cisplatin, paclitaxel, docetaxel, 5-fluorouracil, methotrexate, doxorubicin (DOX), bleomycin, and others are representative drugs used to treat OSCC [4, 5]. Although there have been significant improvements in chemotherapy regimens and administration methods in recent years, the overall survival rate of OSCC has not been significantly improved by chemotherapy. The main reasons include poor water solubility, short half-life, low targeting specificity, and significant toxic side effects of chemotherapy drugs [6]. Therefore, improving the pharmacological properties of drugs and enhancing the clinical efficacy of chemotherapy are urgent tasks that need to be addressed.

With the development of nanotechnology, nanoparticles (NPs) have been widely used in passive targeted therapy for OSCC. NPs passively target tumor tissues through the enhanced permeability and retention (EPR) effect, thereby increasing the local drug concentration while reducing overall adverse reactions [7]. For example, Mohan et al. used PEGylated liposomal nanocarriers to deliver DOX and trans-resveratrol for OSCC treatment [8]. However, immunogenic reactions and low drug delivery efficiency are major drawbacks in the clinical application of nanomaterials [9]. Thus, researchers have gradually shifted their focus to active targeting strategies.

Active targeting strategies are based on surface modifications with functional groups that can recognize overexpressed receptors or antigens on tumor surfaces. In OSCC treatment, widely used molecular markers and receptors include epidermal growth factor receptor [10], folate receptor [11], CD44 [12], and others. For instance, Cheng et al. developed folic acid-modified cisplatin magnetic nanoparticles for OSCC treatment [11]. Despite this, the tumor-active targeting properties of many engineered NPs are not satisfactory, mainly due to off-target effects in the body, active clearance by macrophages, and low immunocompatibility, which limit further biological applications [13]. The “foreign” property greatly hinders the further clinical application of NPs.

The cell membrane-based biomimetic nanotechnology strategy proposed by Zhang and coworkers provides a new opportunity to address the active targeting of nanoparticles [14]. In recent years, biomimetic cell membrane-coated nanoparticles have attracted widespread attention. By combining the synthesized nanoparticles with different types of cells (white blood cells, platelets, red blood cells, etc.), the nanoparticles can mimic many natural properties of the source cell membrane, such as prolonged circulation time, good biocompatibility, and immune evasion [15]. As a result, the nanoparticles can be accepted by the organism, providing effective drug delivery.

Macrophages, a type of leukocyte, act as circulating sentinel cells to phagocytose foreign substances and exert immune functions. Moreover, macrophages can undergo transendothelial migration ability. Long-term chronic inflammation is an important hallmark of malignancies, attracting macrophages and neutrophils to migrate to tumor sites [16]. Macrophages cells (RAW264.7) can bind to cancer cells through their high expression of integrins α4 and β1, and cancer cell with high expression of vascular cell adhesion molecule-1 (VCAM-1), thus promoting macrophage targeting in breast cancer lung metastasis [17]. C-C chemokine ligand 2 (CCL2) secreted by tumor cells and stromata recruits macrophages into the tumor microenvironment through the CCL2-CCR2 mechanism [18, 19]. Nanoparticles coated with macrophages can inherit their tumor targeting ability, immune evasion, and extended circulation time. Recently, this biomimetic strategy has demonstrated unique therapeutic effects in various cancers, such as breast cancer [20], colon cancer [21], and gliomas [22]. For example, Cao and coworkers used macrophage membrane-coated liposomes encapsulating the anticancer drug emtansine to significantly inhibit breast cancer and its lung metastasis [23]. To date, this biomimetic strategy has not been explored in OSCC-related applications. The source of macrophage membrane is currently mainly from RAW264.7, J774A.1, THP-1 and blood or tissues [24, 25]. Coating Tumor-associated macrophage membrane to NPs targeted tumor and switched the activation of macrophages from M2-like phenotype to a more inflammatory M1-like state [25]. M1 macrophages membrane-encapsulated NPs, derived from the RAW264.7 cells upon lipopolysaccharide (LPS) stimulation, enhanced tumor targeting ability [26, 27]. Currently the most used macrophage membranes are derived from RAW264.7, showing immune escape and tumor targeting [20, 23, 28].

As well known that the tumor microenvironment results in decreased pH due to oncogenic transformation and abnormal metabolism [29]. The acidic tumor microenvironment increases the risk of local invasion, metastasis, and therapeutic resistance. However, the environment also provides an opportunity for the application of pH-responsive drugs. For example. DOX was encapsulated into carriers through acylhydrazone linkages, which broke in the tumor microenvironment to achieve drug release and enhance its therapeutic effect on OSCC [30]. It has been reported that certain diols can complex with boronic acid in aqueous solutions by forming reversible boronate ester bonds. Then, boronic acid combines with the diols to form cyclic boronate esters, which dissociate in acidic environments. The stability of boronate ester complexes largely depends on pH [31]. Through this binding, boronic acid can be used as a sensor for sugars [32], as a transporter for nucleotides and carbohydrates [33], and for drug delivery under specific conditions [34].

**Fig. 1:**
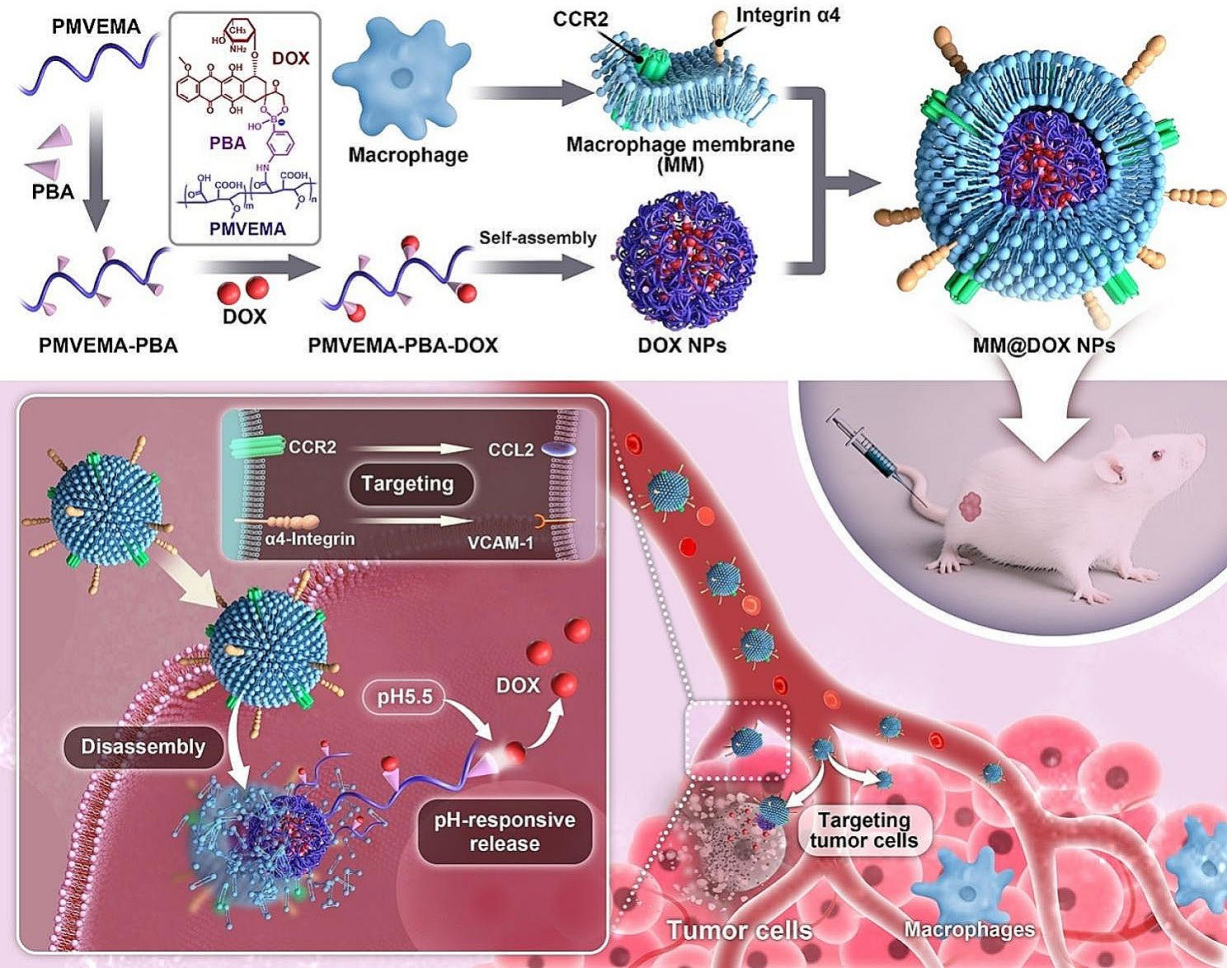
Schematic illustration of the synthesis and antitumor performance of MM@DOX NPs

Therefore, we designed a pH-responsive nanocarrier drug delivery system consisting of macrophage membrane-coated NPs for the targeted treatment of OSCC (Fig. 1). In this study, we grafted phenylboronic acid (PBA) moiety onto the hydrophilic polymer poly(methyl vinyl ether *alt* maleic anhydride) (PMVEMA) to form PMVEMA-PBA. The polymer was further loaded with DOX, resulting in the formation of PMVEMA-PBA-DOX. PMVEMA-PBA-DOX was self-assembled to form nanostructures (DOX NPs). Furthermore, DOX NPs were encapsulated with macrophage membrane (MM) to form MM@DOX NPs. As a result, the nanocarriers could respond to the low pH value in the tumor microenvironment and selectively release the loaded DOX within tumor cells, owing to the boronate ester bond between the PBA and DOX [29, 35, 36]. Furthermore, the enveloped macrophage membrane imparts tumor-targeting, immune evasion abilities and extended circulation time to the nanoparticles. Overall, the aim of this study was to evaluate the delivery capability of this formulation and its therapeutic effects on two different oral squamous cell carcinoma cell lines SCC15 and HN6, as well as an oral squamous cell carcinoma bearing mouse model, providing a reference for the treatment of OSCC.

## Materials and methods

### Materials

Dulbecco’s modified Eagle’s medium (DMEM), fetal bovine serum (FBS), trypsin EDTA, penicillin and streptomycin and phosphate-buffered saline (PBS) were provided by Gibco (Carlsbad, CA). The cell viability/cytotoxicity assay kit, cell counting kit-8 (CCK-8), 4′, 6-diamidino-2-phenylindole (DAPI), BCA Protein Assay Kit, Coomassie brilliant blue, FITC-labelled goat anti-mouse IgG (H + L), chemiluminescence detection kit and radioimmunoprecipitation assay buffer (RIPA) were provided by Beyotime Biotechnology (Shanghai, China). 3-aminophenylboronic acid monohydrate (PBA), poly(methyl vinyl ether-*alt*-maleic anhydride) (PMVEMA), trithylamine, dimethyl sulfoxide (DMSO) and doxorubicin hydrochloride were obtained from Macklin Biochemical (Shanghai, China). DiD was supplied by Biotium Inc (Fremont, US). Anti-integrin α4 monoclonal antibody and anti-CCR2 monoclonal antibody were provided by Abcam (Cambridge, U.K.). Unless otherwise stated, all reagents were of analytical grade and employed without further purification.

### Cell culture

RAW264.7 cells, and the human squamous carcinoma HN6 and SCC15 cells were incubated in DMEM supplemented with 10% FBS, 100 U/mL penicillin, and 100 µg/mL streptomycin. Cells were stored in an incubation chamber at 37 °C and 5% CO_2_ with a humidified atmosphere.

### Synthesis and characterizations of PMVEMA-PBA

In brief, PBA, PMVEMA, and trithylamine were dissolved in DMSO, and stirred at 40 °C for 24 h. The solutions were dialyzed against excess deionized water with a dialysis bag (MWCO: 3.5 kDa) for 6 h. The final product was obtained by lyophilization. The FT-IR spectra of PMVEMA, PBA and PMVEMA-PBA were recorded from 4000 to 400 cm^− 1^ to examine the changes in functional groups. ^1^H-NMR was performed to confirm the modification.

### Synthesis and characterizations of DOX NPs

DOX NPs were synthesized as follows: PMVMA-PBA and DOX were dissolved in DMSO. Trithylamine was added to the solution, and stirred at 40 °C for 24 h. Next, the resulting conjugates were dialyzed against excess deionized water with a dialysis bag (MWCO: 3.5 kDa) for 12 h. The whole reaction was performed away from light. The FT-IR spectra of DOX NPs were recorded from 4000 to 400 cm^− 1^ to examine the changes in functional groups.

### Preparation of MM@DOX NPs

The method was performed according to a previous report [37]. In detail, RAW264.7 cells were seeded onto 10 cm dishes and were left to grow and cover the bottom. The cells were harvested and suspended in hypotonic lysing buffer. The cell suspension was ground for 20 times by using a hand grinder to generate cell fragments. Then the mixture was centrifuged at a gentle rotation speed (3200 g, 5 min) to remove heavy cell organelles, and at a strong rotation speed (20,000 rcf, 20 min) to collect the cell membranes. The cell membranes were resuspended in diethyl pyrocarbonate (DEPC) solution for further use.

The macrophage membrane was coated onto the DOX NPs through the extrusion method. The cell membrane solution was added to the DOX NPs solution, and was sonicated at a power level of 60 W for 5 min. Then the mixture was successively extruded through a polycarbonate membrane with the pore size of 400 nm to form macrophage membrane-coated DOX NPs (MM@DOX NPs).

### Characterization of MM@DOX NPs

To determine the decoration of the macrophage membrane, the hydrodynamic size and zeta potential were measured using a dynamic light scattering (DLS) detector Zetasizer Nano-ZS (Malvern, U.K.). The morphologies at pH 7.4 and 5.5 were characterized under a HT7800 biological transmission electron microscope (TEM) (Hitachi, Japan). Standard curves of DOX were determined by UV-vis spectrophotometry. Drug loading and encapsulation efficiency were calculated through the corresponding standard curve.

### Stability evaluation of MM@DOX NPs

Stability evaluation of MM@DOX NPs was carried out as reported [38]. In brief, MM@DOX NPs were diluted with pH 7.4 or 5.5 PBS at 37 °C. The size distribution and polydispersibility index (PDI) were detected with a Zetasizer Nano-ZS (Malvern, U.K.) over 6 days to evaluate its stability over time.

### Protein detection of RAW264.7 cells and MM@DOX NPs

The protein profiles in macrophages, macrophage membranes and MM@DOX NPs were determined by SDS-PAGE. Proteins were extracted from the macrophages, macrophage membranes and MM@DOX NPs with RIPA lysis buffer. The protein concentrations were quantified by a BCA Protein Assay Kit. Subsequently, the protein was subjected to an electrophoresis assay. The resulting gels were stained with Coomassie brilliant blue for 2 h and washed for 12 h. The treated gels were captured by a ChemiDOC^TM^XRS+System (Bio-rad, USA).

To further characterize the expression of integrin α4 and CCR2 in cell membranes, the resultant gels were transferred to polyvinylidene difluoride (PVDF) membranes for western blot analysis. PVDF membranes were probed with antibodies overnight at 4 °C, after blocking with 5% skim milk. The PVDF membranes were incubated with horseradish peroxidase-conjugated rabbit anti-mouse IgG (H + L) secondary antibody at room temperature for 1.5 h and washed three times. PVDF membranes were visualized with an enhanced chemiluminescence detection kit. The protein signals were captured with a ChemiDOC^TM^XRS+System (Bio-Rad, USA).

### Determination of pH-sensitive drug release

The drug release kinetics of DOX from DOX NPs and MM@DOX NPs were monitored in PBS at two pH values (pH 7.4 and 5.5) by a dialysis method. The drugs were added to the dialysis bag (MWCO: 3.5 kDa) and was dialyzed against corresponding pH value buffers under gentle stirring at 37 °C. At predetermined time intervals, the medium was removed and an equal amount of fresh buffer was replenished. The amount of DOX released from DOX NPs and MM@DOX NPs was detected by UV-visible spectrophotometry at 480 nm.

#### In vitro cellular uptake examined by fluorescence microscopy

To visualize the endocytosis process of nanoparticles by SCC15 and HN6 cells, fluorescence images were captured at 1, 3 and 5 h. SCC15 and HN6 cells were placed into 6-well plates at a density of 1 × 10^5^ cells per well. After the cells were cultured for 24 h, the cell culture media were then replaced with fresh media containing free DOX, DOX NPs and MM@DOX NPs. The cells were incubated for predetermined intervals at 37 °C. Subsequently, the cells were washed twice with PBS and fixed with paraformaldehyde (PFM) for 20 min. DAPI was added for nuclear staining. The cells were rinsed three times with PBS. Then fluorescence images were captured by an EVOS FL Auto fluorescence microscope (Thermo Fisher, USA).

#### In vitro cellular uptake examined by flow cytometry

To quantitatively measure intracellular uptake, the cell uptake behaviour of free DOX, DOX NPs and MM@DOX NPs were investigated by flow cytometry. SCC15 and HN6 cells were placed into 6-well plates at a density of 1 × 10^5^ cells per well. After culturing for 24 h, the cells in each well were treated with drugs for 1, 3 and 5 h, respectively. The cells were harvested by trypsin treatment at the indicated time points after they were washed three times with cold PBS, and suspended in PBS. The data was detected by flow cytometry (Beckman Coulter, USA) and FLOWJO 10 software.

### Immune escape characteristics

To determine the immune escape abilities of the resulting nanoparticles in vitro, fluorescence microscopy and flow cytometry were used to evaluate macrophage uptake of different nanoparticles. RAW264.7 cells were seeded into 6-well plates at a density of 1.0 × 10^5^ cells per well. Incubation was performed at 37 °C for 24 h. Cell culture media were then replaced with fresh media containing free DOX, DOX NPs and MM@DOX NPs. After 1, 3, and 5 h, the cells were washed with PBS, fixed with PFM for 20 min, labelled with DAPI for 15 min, and then rinsed three times with PBS. Then the cells were observed using a fluorescence microscope. For quantitative measurement, adherent cells were trypsinized into the single cell suspension, suspended in PBS and subjected to flow cytometry.

#### Cytotoxicity and antitumor efficacies in vitro

The in vitro cytotoxicity of free DOX, DOX NPs and MM@DOX NPs were assessed in SCC15 and HN6 cells through a CCK-8 assay. A CCK-8 assay was also conducted under pH 6.5 conditions in HN6 cells. Briefly, 1.0 × 10^4^ cells were added to each well in 96-well plates. After 24 h, free DOX, DOX NPs and MM@DOX NPs were respectively added to each well at different concentrations ranging from 0.156 µg /mL to 20 µg/mL using media at different pH values. The nontreated cells were used as a negative control. After 24 h of incubation, the medium was discarded, and CCK-8 solution was added to each well for another 1–4 h. The absorbency of each well was measured using a multifunctional microporous plate reader SpectraMAX iD5 (Molecular Devices, USA) at a wavelength of 450 nm, and cell survival was calculated. The half-inhibitory concentration (IC_50_)was calculated.

### Live/dead cell assay

To assess the in vitro therapeutic effect of different nanoparticles on SCC15 and HN6 cells, a cell viability/cytotoxicity assay kit was adopted, which could label live/dead cells with calcein-AM/propidium iodide (PI) probes. SCC15 and HN6 cells were respectively placed into 6-well plates at a density of 1 × 10^5^ cells per well. After incubation for 24 h at 37 °C, free DOX, DOX NPs and MM@DOX NPs were added to each well. After 24 h, the cells were incubated with calcein-AM/PI double staining kit for 30 min at 37 °C in the dark. The staining effect was observed by fluorescence microscopy. Finally, the cells were harvested and analysed by flow cytometry.

### Animals use and care

Male BALB/c mice were purchased from the animal centre of Chongqing Medical University. Experiments were performed with permission from the Ethics Committee of the College of Stomatology, Chongqing Medical University. The mice were randomly allocated into each group.

#### Biodistribution in vivo

The in vivo systematic circulation of the nanoparticles was measured by small animal in vivo imaging systems. DiD, a lipophilic near-infrared fluorescent dye, was used to mark different nanoparticles [39]. Tumor-bearing mice were intravenously injected with DiD-loaded nanoparticles via the tail vein at a DiD dose (2 mg/kg). Fluorescence imaging was visualized at 24 h. The tumor and major organs (heart, liver, spleen, lung and kidney) were harvested after 24 h. The DiD fluorescence intensity in excised organs and tumor was examined using an imaging system (Xenogen, USA).

To further evaluate targeting-ability of MM@DOX NPs in vivo, the tumor tissues were harvested, sectioned and immunostained with CCR2 or integrin α4 polyclonal antibody and FITC-labelled goat anti-mouse IgG (H + L) after 24 h post-injection of free DiD, DiD NPs and MM@DiD NPs. Then, the fluorescent co-localization of DiD and CCR2 or integrin α4 was observed by fluorescence microscopy.

#### Pharmacokinetics in vivo

BALB/c mice were intravenously injected with DiD-loaded nanoparticles via the tail vein at a DiD dose (2 mg/kg). At different time points (0.01, 1, 4, 8, 12, 24, 48 h), the blood samples were collected from the orbital vein in heparin sodium-containing tubes, and stored at 4 °C. The blood samples were transferred to 96-well plates. The DiD fluorescence intensity of blood samples was examined using a microplate apparatus (Tecan, USA).

#### In vivo antitumor effect

HN6 cells were subcutaneously injected into BALB/c mice (male, 4–6 weeks) at a density of 1 × 10^6^ cells to develop the HN6 tumor-bearing BALB/c mouse model. The mice were randomly separated into four groups (*n* = 4 in each group) and dosed for 14 days by tail vein injection every two days. In the treatment groups, mice were administered: PBS, free DOX, DOX NPs or MM@DOX NPs at a dose of 1 mg/kg. The body weight and tumor volume were measured every other day. Two weeks later, the mice were euthanized. Before sacrifice, blood samples were collected from the orbital vein before sacrifice and analysed at the Stomatological Hospital of Chongqing Medical University, including the levels of alanine transaminase (ALT), aspartate transaminase (AST), creatinine (CREA) and blood urea nitrogen (BUN). The major organs, including the heart, liver, spleen, lung, and kidney, were collected and fixed with 4% PFM for further H&E staining.

### Hemolysis assay

A hemolysis experiment was performed to evaluate the safety of nanoparticles. Briefly, fresh BALB/c blood was obtained in heparin sodium-containing tubes. The fresh blood was centrifuged, and red blood cells (RBCs) were obtained. The RBCs were then resuspended in saline. Free DOX, DOX NPs and MM@DOX NPs were separately added to the RBC suspension at final concentrations of 0.312, 0.625, 1.25, 2.5, 5, 10 and 20 µg/mL. The RBC suspension was diluted with saline as the negative control and with deionized water as the positive control. After incubation for 4 h at 37 °C, the suspension was centrifuged and photographed. The hemolysis rate was then measured and calculated.

### Statistical analysis

The results were expressed as the mean ± standard deviation (SD). Statistical analysis was performed using SPSS 21.0 software. The data were subjected to one-way ANOVA analysis for multiple groups. The statistical significance levels are represented as ^*^ for *p* < 0.05, ^**^ for *p* < 0.01, and ^***^ for *p* < 0.001.

## Results and discussion

### Synthesis and characterization of MM@DOX NPs

**Fig. 2:**
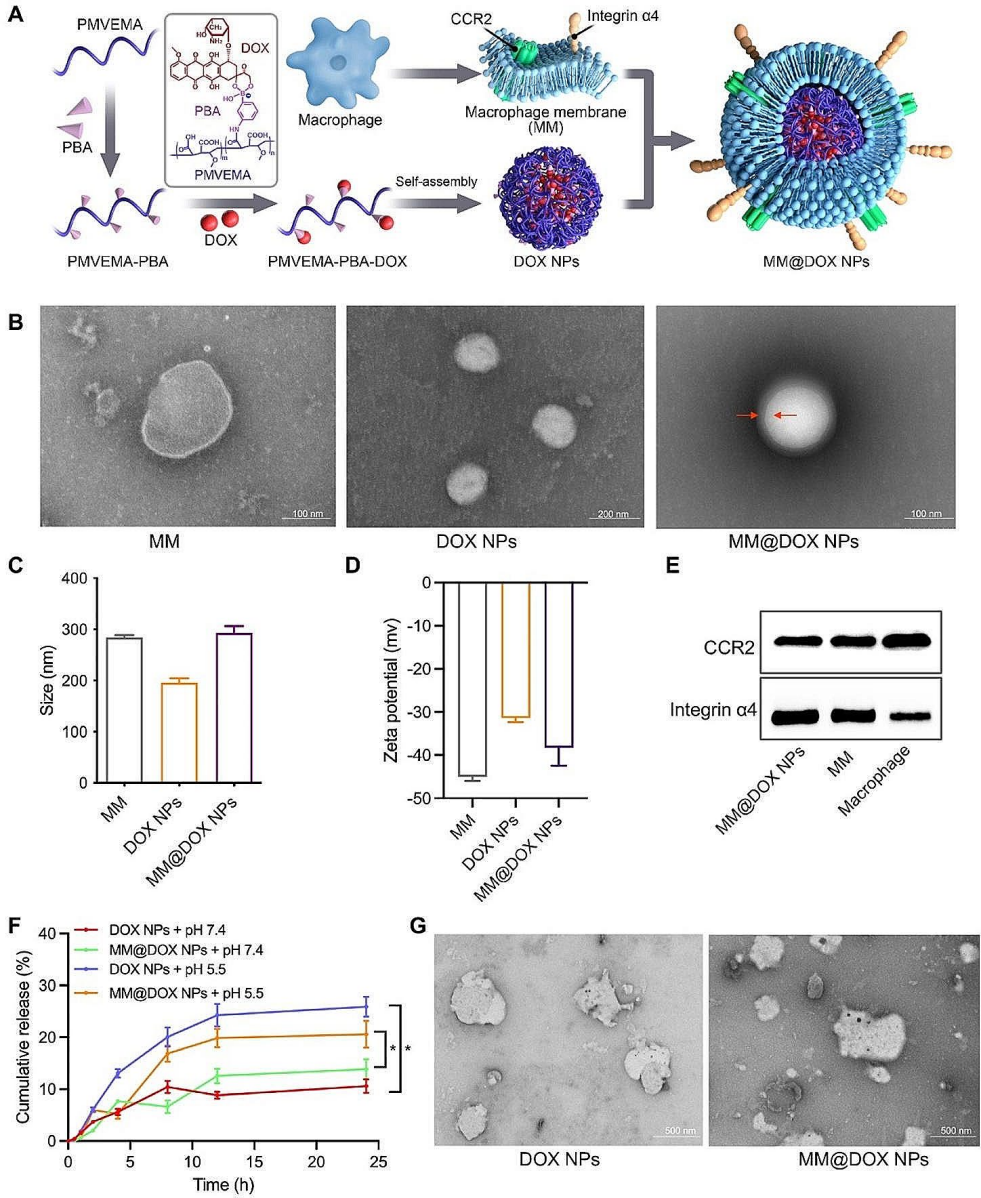
**A** Preparation process of MM@DOX NPs drug delivery system. **B** TEM image of MM, DOX NPs and MM@DOX NPs (the red arrow showed the cell envelope). **C** The hydrodynamic size and **D** zeta potential of MM, DOX NPs and MM@DOX NPs (*n* = 3, mean ± SD). **E** Western blot analysis of CCR2 and integrin α4 in MM@DOX NPs, MM and macrophage. **F **In vitro drug release study with DOX NPs and MM@DOX NPs in PBS under different pH values (*n* = 3, mean ± SD). **G** The morphology of DOX NPs and MM@DOX NPs after incubation at pH 5.5. (* *p* < 0.05)

The route of MM@DOX NPs synthesis was depicted in Fig. 2A. First, PMVEMA-PBA was prepared by the ring opening reaction between PMVEMA and PBA. The structure was characterized by FT-IR and ^1^H-NMR (Figs. S1,S2). From the FT-IR results (Fig. S1), it could be observed that the C = O stretching absorption peaks at 1856 cm^− 1^ and 1780 cm^− 1^ gradually weaken. This was due to the esterification reaction between PMVEMA and PBA. The two conjugated carbonyl groups of maleic anhydride were opened to form one ester carbonyl group and one carboxylic acid carbonyl group, resulting in a new C = O stretching absorption peak at 1731 cm^− 1^. Additionally, a new absorption peak appeared at 1558 cm^− 1^, which was attributed to the C = C structure introduced in phenylboronic acid. The appearance of these aforementioned features indicated that PMVEMA-PBA was successfully synthesized. Then, DOX, a chemical antitumor drug, was loaded onto PMVEMA-PBA by simple mixing. Significant absorptions were observed at approximately 1578 cm^− 1^, 1213 cm^− 1^, and 1085 cm^− 1^, which were mainly caused by the introduction of the doxorubicin structure, leading to the formation of triangular-coordinate borate compounds and tetrahedral-coordinate borate compounds. In addition, the enhanced absorption at 1000 cm^− 1^ indicated that several structures, such as the anthracene ring in doxorubicin, were introduced, confirming the successful synthesis of PMVEMA-PBA-DOX. Then, PMVEMA-PBA-DOX self-assembled to form DOX NPs.

Macrophage membranes were obtained according to a previous report [37]. RAW264.7 macrophages were disrupted using the direct extrusion method, and the macrophage membranes were isolated by centrifugation. The purified macrophage membranes were mixed with DOX NPs and coextruded to prepare MM@DOX NPs [22]. The loading efficiency and encapsulation efficiency of DOX were calculated to be 9.1% and 54.6%, respectively.

The decoration of macrophage membranes on DOX NPs was determined by various in vitro physicochemical characterizations. The TEM results showed that DOX NPs exhibited a spherical shape, while MM@DOX NPs showed a clear core-shell structure, indicating that encapsulation of DOX NPs by macrophage membranes was successful (Fig. 2B). The average hydrodynamic diameter of MM@DOX NPs in PBS was determined to be 292.7 nm with a narrow size distribution, as determined by DLS measurement, higher than the 195.7 nm of DOX NPs (Fig. 2C, S3). In nanocarriers encapsulated with cell membranes, the surface potential of membrane-modified nanocarriers was close to that of the cell membrane [40, 41]. The zeta potential of MM@DOX NPs was -40.8 ± 3.31 mv, which was comparable to the zeta potential of macrophage membranes (-44.47 ± 0.84 mv) (Fig. 2D). The stability of MM@DOX NPs incubated with PBS for six days was examined by DLS. As shown in the Fig. S4A, the size and PDI of MM@DOX NPs under pH 7.4 showed no significant changes (*p* > 0.05) during 6 days, indicating the sufficient stability for further store and application. While, under acidic condition, the particle size and PDI significantly increased after Day 2 (Fig. S4B), indicating that the MM@DOX NPs were unstable and irregularly aggregated. SDS-PAGE gel electrophoresis was used to analyse the protein profiles of macrophage membranes and MM@DOX NPs. Most of the protein components in macrophage membranes were retained on MM@DOX NPs (Fig. S5). Due to the membrane’s ability to retain glycoproteins, lipids, and proteins on the surface of the cell membrane, the nanoparticles possess functions and characteristics inherent to the source cell. For example, macrophage membrane-mediated tumor targeting via integrin α4 and CCR2 expression [17–19]. To further confirm the potential tumor-targeting effect of MM@DOX NPs, western blotting was performed to detect the expression of integrin α4 and CCR2 on macrophage membranes and MM@DOX NPs, validating that MM@DOX NPs were successfully decorated with macrophage membranes. The specific protein signals of integrin α4 and CCR2 could be observed in macrophage cells, macrophage cell membranes, and MM@DOX NPs, indicating the presence of these protein markers (Fig. 2E). Overall, these evidences suggested that macrophage membranes were successfully modified on DOX NPs.

#### In vitro acid-triggered drug release study

Under acidic conditions, the boronate ester bond is unstable, leading to drug release [29, 35, 36]. Boronate ester bonds between PBA and DOX, exhibit pH sensitivity. In vitro drug release experiments were performed to investigate the pH-sensitive release of nanoparticles. The in vitro release profiles of DOX in DOX NPs and MM@DOX NPs were investigated under acidic condition (pH 5.5) and physiological condition (pH 7.4). The DOX NPs and MM@DOX NPs groups all exhibited burst release within the first 5 h of the experiment, followed by a stable release after 12 h (Fig. 2F). These results indicated that the coating of macrophage membranes and fusion with nanoparticles did not alter the release characteristics of DOX. The formation of boronate ester bonds between PBA and DOX underwent specific hydrolysis under acidic conditions, resulting in nanoparticle degradation and accelerated drug release [42]. Under acidic conditions, the release of DOX in the two groups was higher than that under physiological conditions (*p* < 0.05) (Fig. 2F). The 24 h release of DOX from DOX NPs was higher than that of MM@DOX NPs. MM encapsulation system slowed the release of DOX and contributed to a sustained and orderly release of DOX. These results indicated that MM@DOX NPs had the function of pH-responsive drug release. In addition, as shown in Fig. 2G, the morphology of the nanoparticles was disintegrated and irregularly aggregated under acidic conditions. These results suggested that the designed nanoparticles may provide a drug delivery platform for therapeutic agent specific release in an acidic tumor microenvironment.

#### Targeted characteristics study of MM@ DOX NPs in vitro

**Fig. 3:**
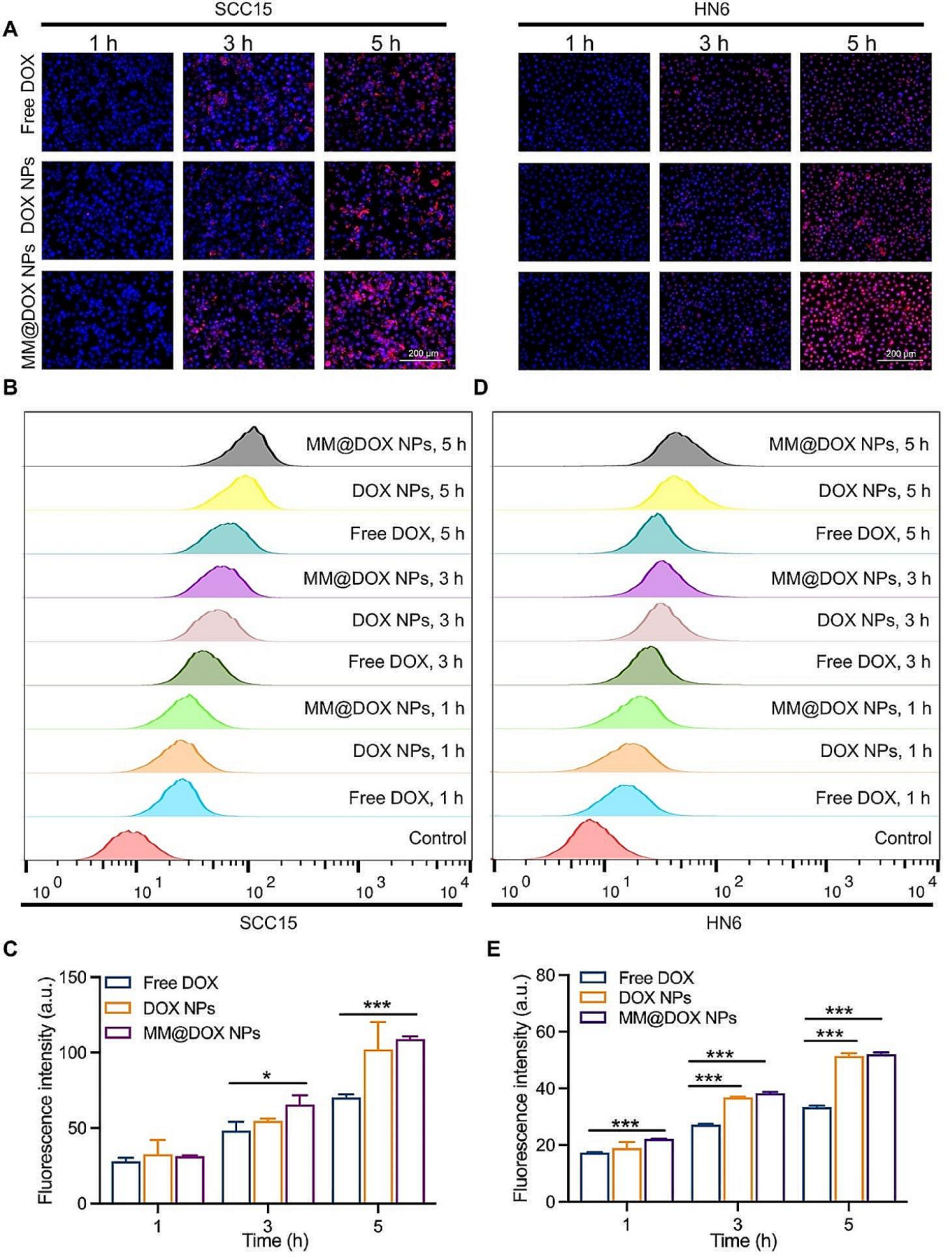
**A** Cellular uptake and localization of free DOX, DOX NPs and MM@DOX NPs in SCC15 or HN6 cells were observed by fluorescence microscopy after incubation of 1, 3 and 5 h, respectively. **B** The flow cytometry profiles incubated with varied treatments in SCC15 for different time intervals and **C** the corresponding quantitative analysis of mean fluorescence intensity (*n* = 3, mean ± SD, * *p* < 0.05, *** *p* < 0.001). **D** The flow cytometry profiles incubated with varied treatments in HN6 for different time intervals and **E** the corresponding quantitative analysis of mean fluorescence intensity (*n* = 3, mean ± SD, *** *p* < 0.001)

Macrophages can be recruited by cancer cells via the CCR2-CCL2 axis, which confers natural tumor targeting to macrophage [18]. It has been reported that a drug delivery platform coated with macrophage membranes has excellent cancer-targeting ability [43]. To verify the targeting ability of MM@DOX NPs, HN6 or SCC15 cells were cocultured with different formulations. The cells were observed using fluorescence microscopy after pretreatment, with DOX’s intrinsic red fluorescence and DAPI’s blue fluorescence for nuclear labelling. As shown in Fig. 3A, for all the formulations, a stronger red fluorescence signal was achieved with 5 h incubation compared to 1 h incubation, indicating that all formulations were internalized by SCC15 and HN6 cells in a time-dependent manner. Additionally, for a certain time of incubation, MM@DOX NPs were internalized more by SCC15 and HN6 cells than DOX NPs and free DOX, demonstrating that the cell affinity of the macrophage membrane was conducive to cell uptake, which was consistent with others’ work [37]. Red fluorescence signals were found in the cytoplasm or nucleus of the cells after 5 h of incubation. In SCC15 cells, fluorescence images acquired after incubation for 5 h showed that the red fluorescence signals in the cytoplasm were stronger than those in the cell nucleus. However, in HN6 cells, the red fluorescence intensity was stronger in the nucleus than the cytoplasm. Free DOX is primarily distributed in the cell nucleus and exerts its drug effect by embedding in DNA and inhibiting macromolecular biosynthesis [44, 45]. This result indicated that NPs were internalized into the nucleus faster in HN6 cells than in SCC15 cells.

Flow cytometry was further used to quantitatively analyse the uptake of the nanoparticles by SCC15 and HN6 cells (Fig. 3B-E). The results showed that SCC15 cells and HN6 cells had the higher uptake of MM@DOX NPs, than the free DOX and DOX NPs groups. Similar results were obtained from the flow cytometry analysis and fluorescence images, demonstrating the internalization-promoted capacity of membrane covering. Notably, after HN6 cells were incubated with the DOX NPs for 3 h and 5 h, the fluorescence intensity was significantly higher than that of the corresponding free DOX groups (Fig. 3E). This result indicated that both DOX NPs and MM@DOX NPs could be effectively taken up by HN6 cells. The efficient internalization of MM@DOX NPs by SCC15 cells and HN6 cells may be attributed to the tumor targeting abilities derived from macrophage membranes as well as to the small hydrodynamic volume [43]. Additionally, PBA could act as a targeting ligand for tumors, facilitating tumor homing, which resulted in the efficient uptake of DOX NPs [46–48].

#### Immune escape characteristics of MM@DOX NPs in vitro

**Fig. 4:**
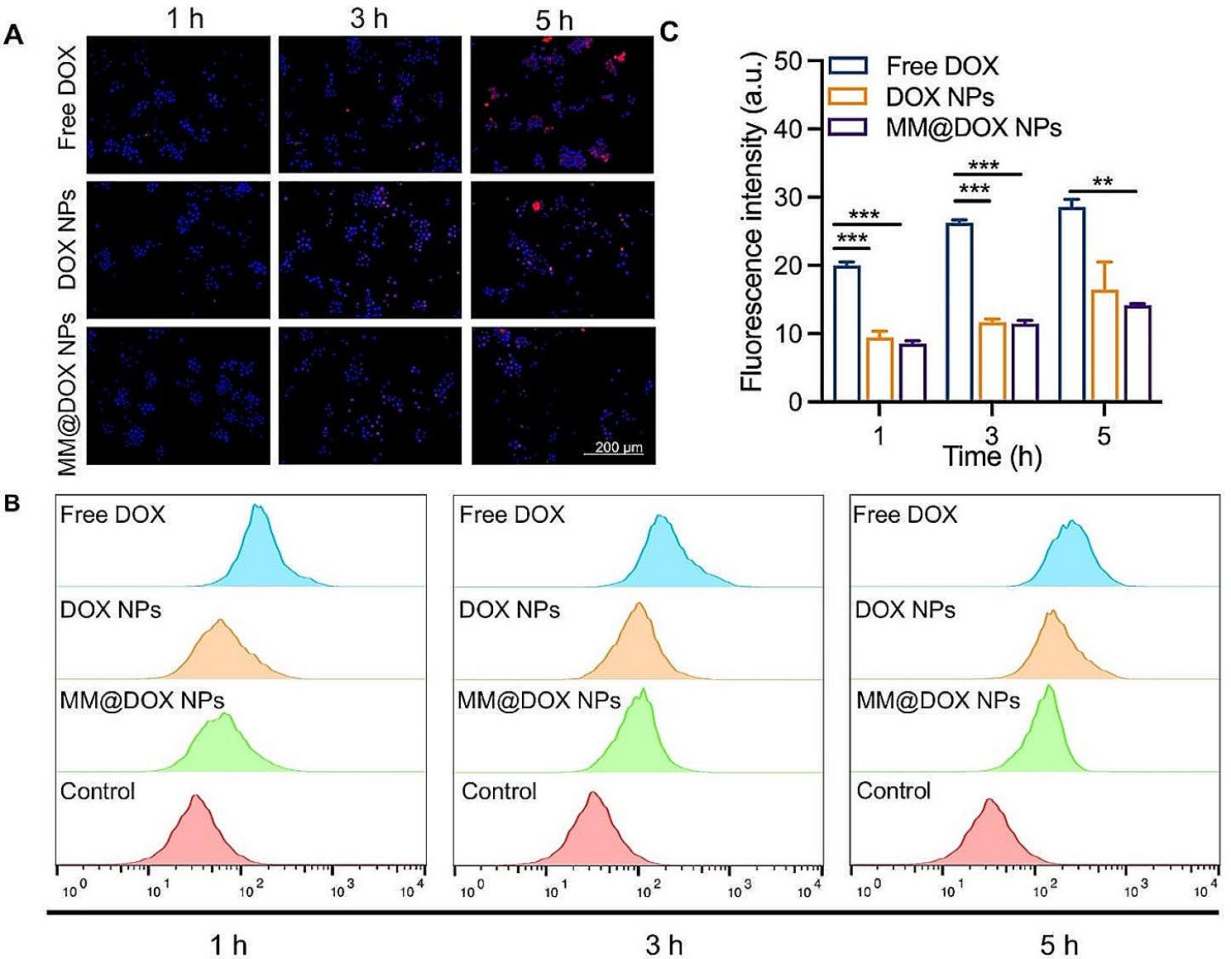
**A** Cellular uptake and localization of free DOX, DOX NPs and MM@DOX NPs in RAW264.7 cells observed by fluorescence microscopy after incubation different time. **B** The flow cytometry profiles of RAW264.7 cells incubated with varied treatments for different time intervals and **C** the corresponding quantitative analysis of mean fluorescence intensity (*n* = 3, mean ± SD, ** *p* < 0.05, *** *p* < 0.001)

To evaluate the immune escape ability of the nanoparticles, the internalization ability of free DOX, DOX NPs, and MM@DOX NPs in macrophages were examined using fluorescent images. The macrophages treated with MM@DOX NPs exhibited the weakest red fluorescence (Fig. 4A). Quantitative analysis using flow cytometry showed that the fluorescence intensity increased with time for all groups (Fig. 4B). After 1, 3 and 5 h of incubation, the fluorescence intensity of the free DOX group was more than twice that of the MM@DOX NPs group (Fig. 4C), indicating that MM@DOX NPs could avoid macrophage phagocytosis. These findings implied that the macrophage membrane endowed the DOX NPs with immune escape abilities to prevent macrophage phagocytosis, which was similar to results recently reported for camouflaged nanocarriers [43].

#### Anticancer assay in vitro

**Fig. 5:**
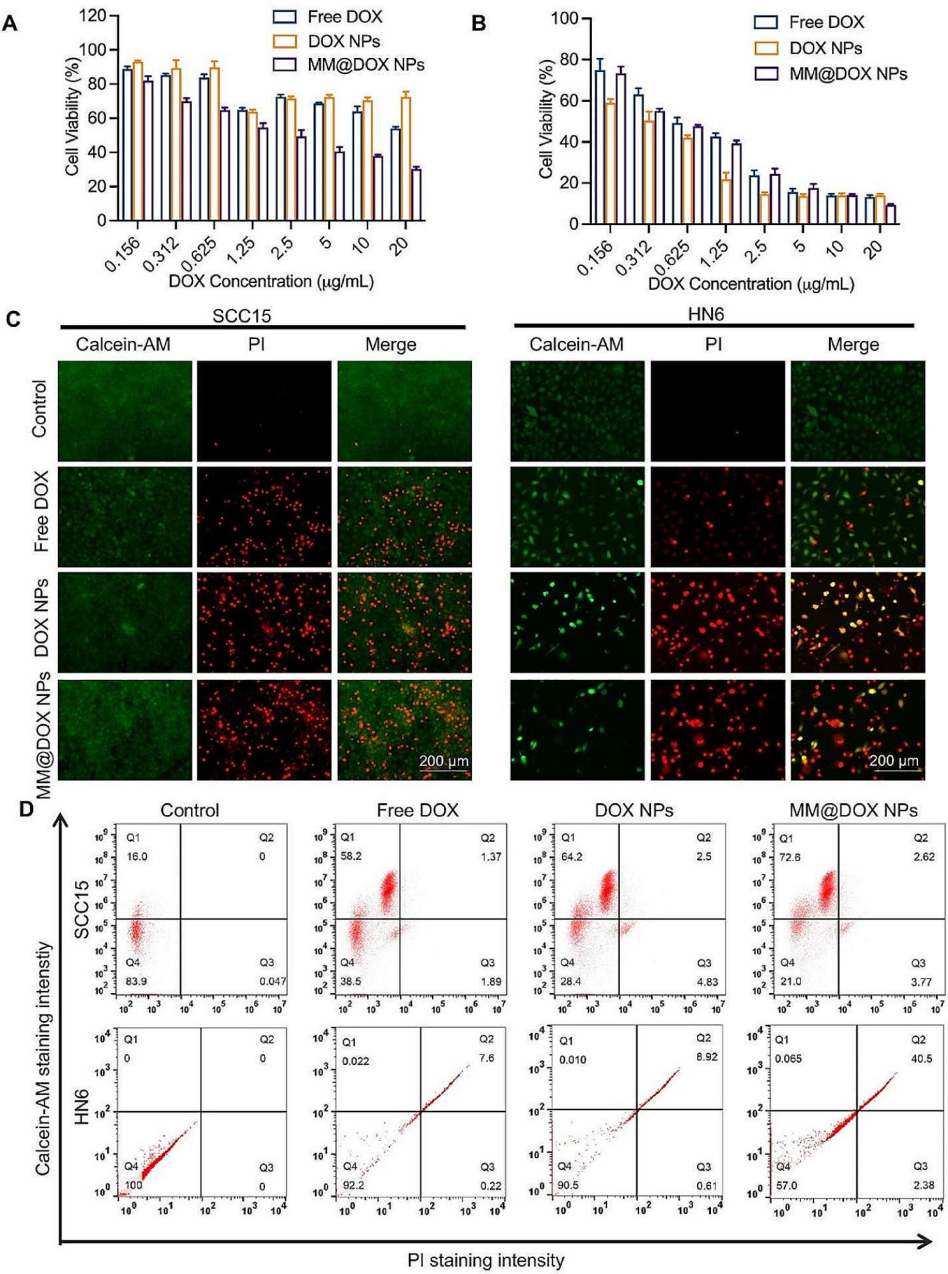
**A** Cell viability of SCC15 cells incubated with free DOX, DOX NPs and MM@DOX NPs at different concentrations (*n* = 4, mean ± SD). **B** Cell viability of HN6 cells incubated with free DOX, DOX NPs and MM@DOX NPs at different concentrations (*n* = 4, mean ± SD). **C** The live/dead staining of SCC15 or HN6 cells incubated with different treatments observed by fluorescence microscopy and **D** the corresponding flow cytometry profiles

To verify the enhanced anticancer effect of MM@DOX NPs, the proliferation inhibition of MM@DOX NPs was tested through a CCK-8 assay against HN6 and SCC15 cells *in vitro.* As shown in Fig. 5A and Fig. 5B the drug inhibition of proliferation varied for different cancer cells. For HN6 cells, the toxicity ranking of the drug was as follows: MM@DOX NPs (IC_50_: 2.825 µg/mL) > DOX NPs (IC_50_: 3.243 µg/mL) > free DOX (IC_50_: 3.846 µg/mL). For SCC15 cells, the IC_50_ value of MM@DOX NPs was 5.867 µg/mL, lower than that of DOX NPs and free DOX. For the two cell lines, the IC_50_ value was lower for MM@DOX NPs than free DOX and DOX NPs. Possibly because MM@DOX NPs were disassembled after internalization by cancer cells. The DOX released by MM@DOX NPs accumulated continuously in tumor cells, thus playing a role in killing cancer cells. Comparing the two cancer cell lines, different OSCC cell lines exhibited varied sensitivities to drugs. MM@DOX NPs showed a stronger tumor cell killing effect on HN6 cells.

Moreover, calcein-AM/PI staining further confirmed the effect of MM@DOX NPs on tumor cells. Cells in green are live cells, while those in red are dead cells. In SCC15 cells, green fluorescence was observed in each group (Fig. 5C). Comparable results were detected in groups of MM@DOX NPs in HN6 cells, in which little green fluorescence was found. Compared with free DOX, MM@DOX NPs exhibited a significant red fluorescence signal in both cell lines. Therefore, the MM@DOX NPs achieved outstanding therapeutic effects. Flow cytometry was further used to quantitatively analyse the dead cells after treatments (Fig. 5D). More dead cells were observed when SCC15 cells and HN6 cells were incubated with MM@DOX NPs than the free DOX group. Compared to SCC15 cells, HN6 cells treated with MM@DOX NPs contained more dead cells and fewer living cells. The maximum of approximately 42.88% dead cells was observed in HN6 cells treated with MM@DOX NPs. These results indicated that MM@DOX NPs could produce significant cytotoxic effects on SCC15 cells and HN6 cells. Moreover, MM@DOX NPs had a stronger effect on HN6 cells.

To study the effects of the drug on cancer cells under acidic conditions, a CCK-8 assay was conducted at pH 6.5 conditions to evaluate the cytotoxicity (Fig. S6). The results showed that under acidic conditions, for HN6 cells, the toxicity rankings of drugs were as follows: DOX NPs (IC_50_: 0.23 µg/mL) > MM@DOX NPs (IC_50_: 0.43 µg/mL) > free DOX (IC_50_: 1.87 µg/mL). In the acidic environment, the IC_50_ values of both MM@DOX NPs and DOX NPs significantly decreased, indicating higher cell toxicity under acidic conditions. The increase in drug release at lower pH enhanced their effects. The presence of the membrane hindered drug release, resulting in lower cytotoxicity for MM@DOX NPs compared to DOX NPs. However, the cytotoxicity was still higher than that of free DOX.

#### Biodistribution in vivo

**Fig. 6:**
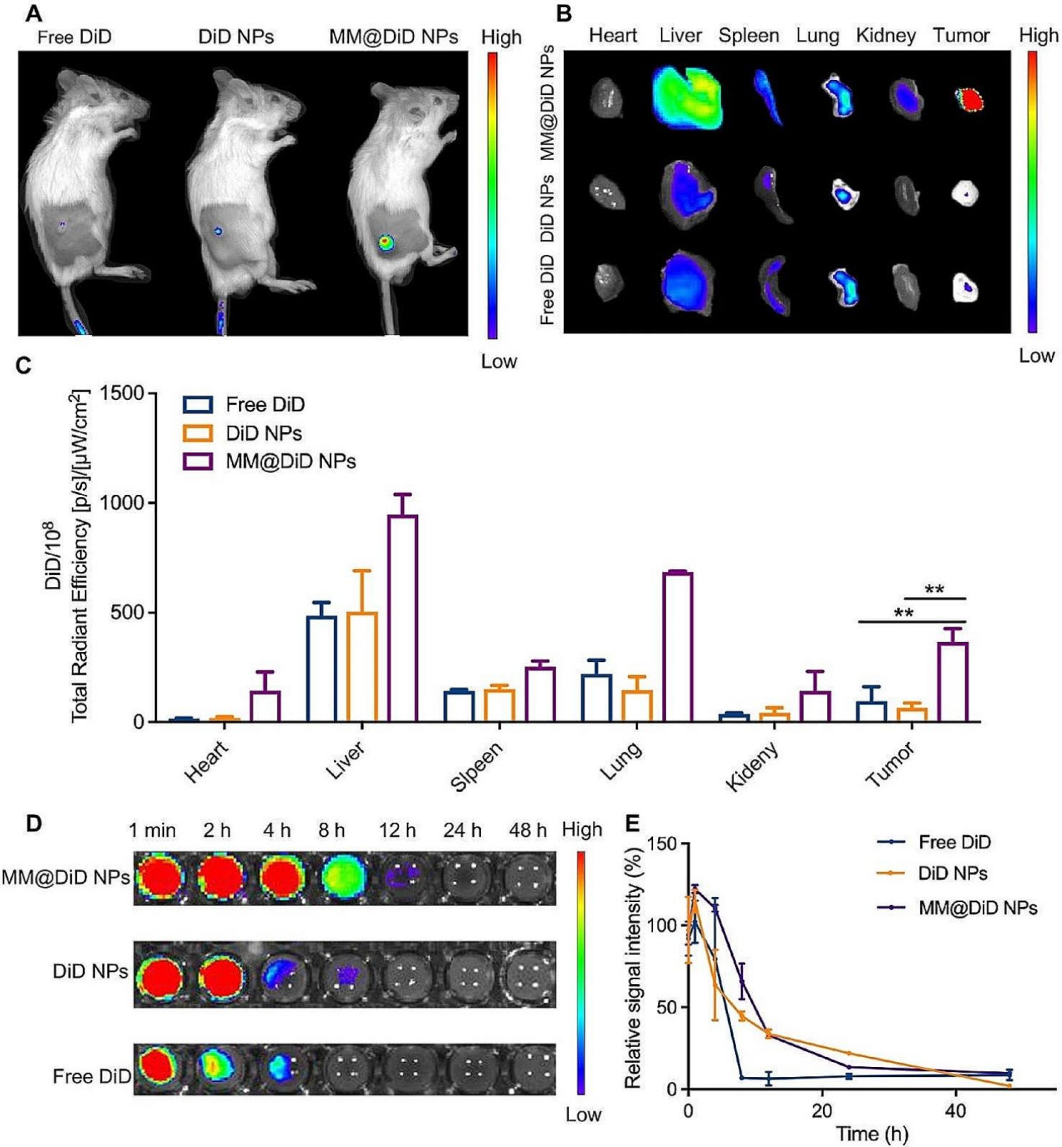
**A **In vivo imaging of the mice after free DiD, DiD NPs and MM@DiD NPs injection at 24 h. **B** Ex *vivo* organ imaging of mice and **C** quantitative fluorescence intensity of the main organs in ex *vivo* organs (*n* = 3, mean ± SD, ** *p* < 0.01). **D** Blood fluorescence images of free DiD, DiD NPs and MM@DiD NPs over a span of 48 h after injection, and **E** corresponding fluorescence intensity (*n* = 3, mean ± SD)

Macrophages have tumor targeting ability through the α4β1/VCAM-1 interaction, and CCL2 secreted in the tumor microenvironment can recruit more macrophages [18]. To evaluate whether macrophage membrane-coated nanoparticles could enhance the targeting ability of nanoparticles, we used an HN6-bearing BALB/c mouse model for in vivo fluorescence imaging. MM@DiD NPs showed evident tumor accumulation 24 h after intravenous injection, with significantly higher fluorescence intensity than that of DiD NPs and free DiD (Fig. 6A). Therefore, macrophage membrane camouflage was successful with strong recruitment ability and positive tumor targeting of MM@DiD NPs. The ex vivo fluorescence images of major organs (heart, liver, spleen, lung, kidney) and tumors of the three groups 24 h after intravenous injection were displayed in Fig. 6B. Compared with the other two groups, the fluorescence intensity of MM@DiD NPs in the tumor was significantly higher (Fig. 6C), which corresponded with the in vivo outcome. This result demonstrated that the macrophage membrane benefited the tumor distribution, which corresponded with other findings [49].

After 24 h of injection, the spatial distribution of nanoparticles was determined (Fig. S7). The weak fluorescence of DiD NPs and free DiD was due to the immune clearance. However, MM@DiD NPs accumulated at the tumor site with the higher DiD fluorescence intensity, compared with the other two groups (*p* < 0.05), and penetrated into the interior areas of the tumor mass, which promoted significant local drug delivery. Various chemokines, including α4 and β1 integrins, MAC-1 and CCR2, served as potent chemo-attracts for the recruitment of macrophages to the tumor site [28, 50, 51]. Not only the high intensity of DiD at tumor site in MM@DiD NPs, but the immunofluorescence of CCR2 and integrin α4 could also been seen in the tumor site. On all these counts, MM@DiD NPs inherited the unique homing biological function from macrophages and improved the targeting efficiency, resulting in significant NPs accumulation at the tumor site.

#### Pharmacokinetics in vivo

To further study in vivo pharmacokinetics, the blood distribution of MM@DiD NPs with time after a single intravenous injection was investigated (Fig. 6D-E). Free DiD was rapidly cleared from the bloodstream. In contrast, the elimination half-lives of MM@DiD NPs and DiD NPs were extended to approximately 9.26 h and 7.53 h, respectively, which was much longer than the 1.94 h half-life of free DiD. This finding was consistent with the in vitro immune escape experiment. The results indicated that the half-lives of nanoparticles coated with the macrophage membrane were extended, effectively avoiding the immune system clearance [52].

#### In vivo antitumor effects on HN6 tumor-bearing mice

**Fig. 7:**
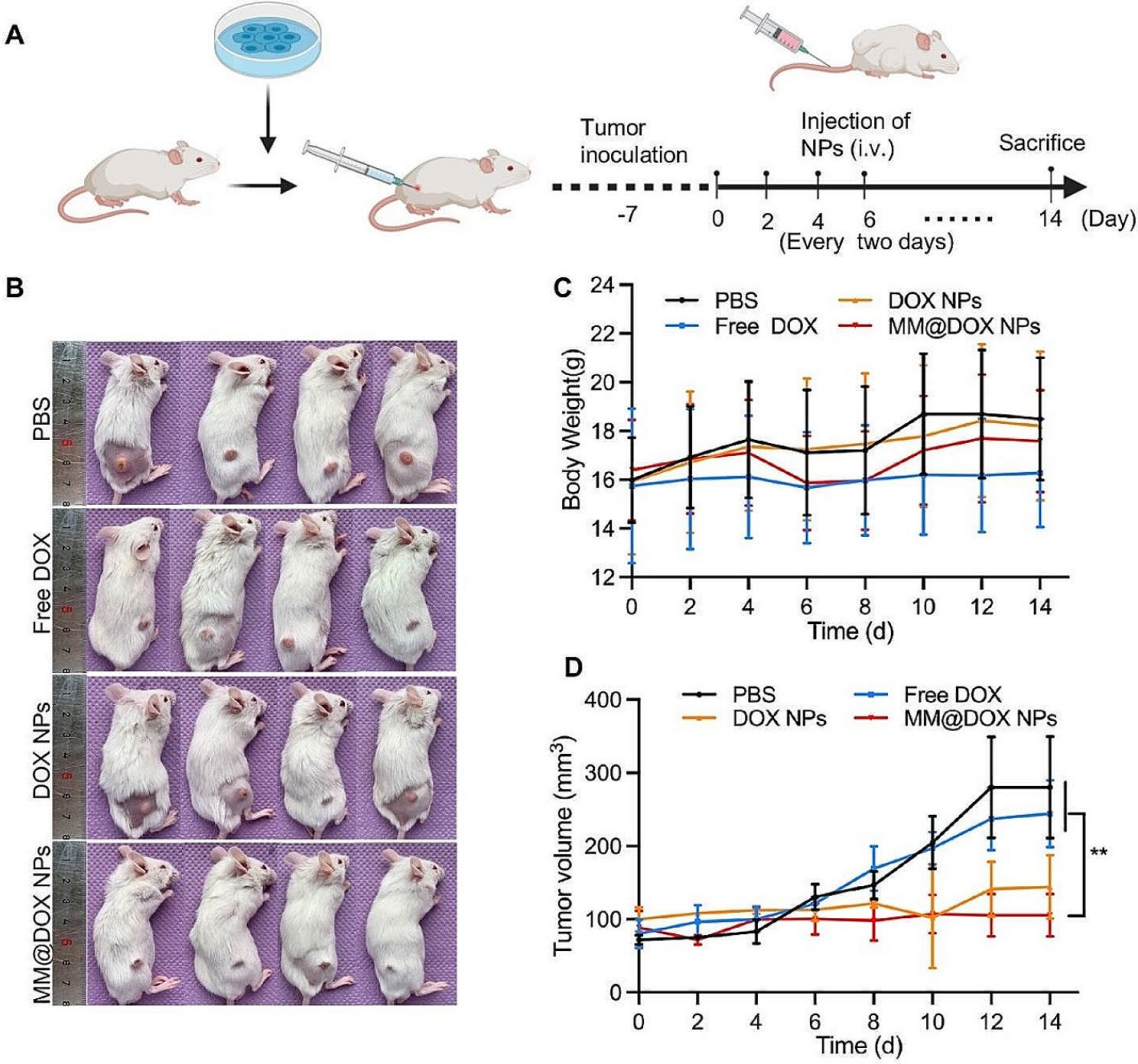
**A** Schematic illustration of various cancer treatments of HN6 tumor-bearing mice. **B** Photos of HN6 tumor-bearing mice. **C** Body weight changes and **D** tumor size changes in HN6-tumor-bearing mice with various treatments (*n* = 4, mean ± SD)

To evaluate the anticancer effect of MM@DOX NPs in oral squamous cell carcinoma xenograft mice, mice were intravenously injected with different formulations 7 times, every 2 days. The treatment plan was shown in the schematic diagram in Fig. 7A. Mouse weight and tumor volume were recorded every 2 days to assess general toxicity and antitumor activity (Fig. 7B-D). The PBS-treated group showed no effect on OSCC proliferation, which presented a much larger tumor volume on Day 14 than on Day 0. In general, after successive treatment, various treatment groups showed differences in tumor suppression. Notably, tumor burden was significantly controlled in mice treated with MM@DOX NPs, and a healthy body weight was maintained.

### Biosafety of MM@DOX NPs

**Fig. 8:**
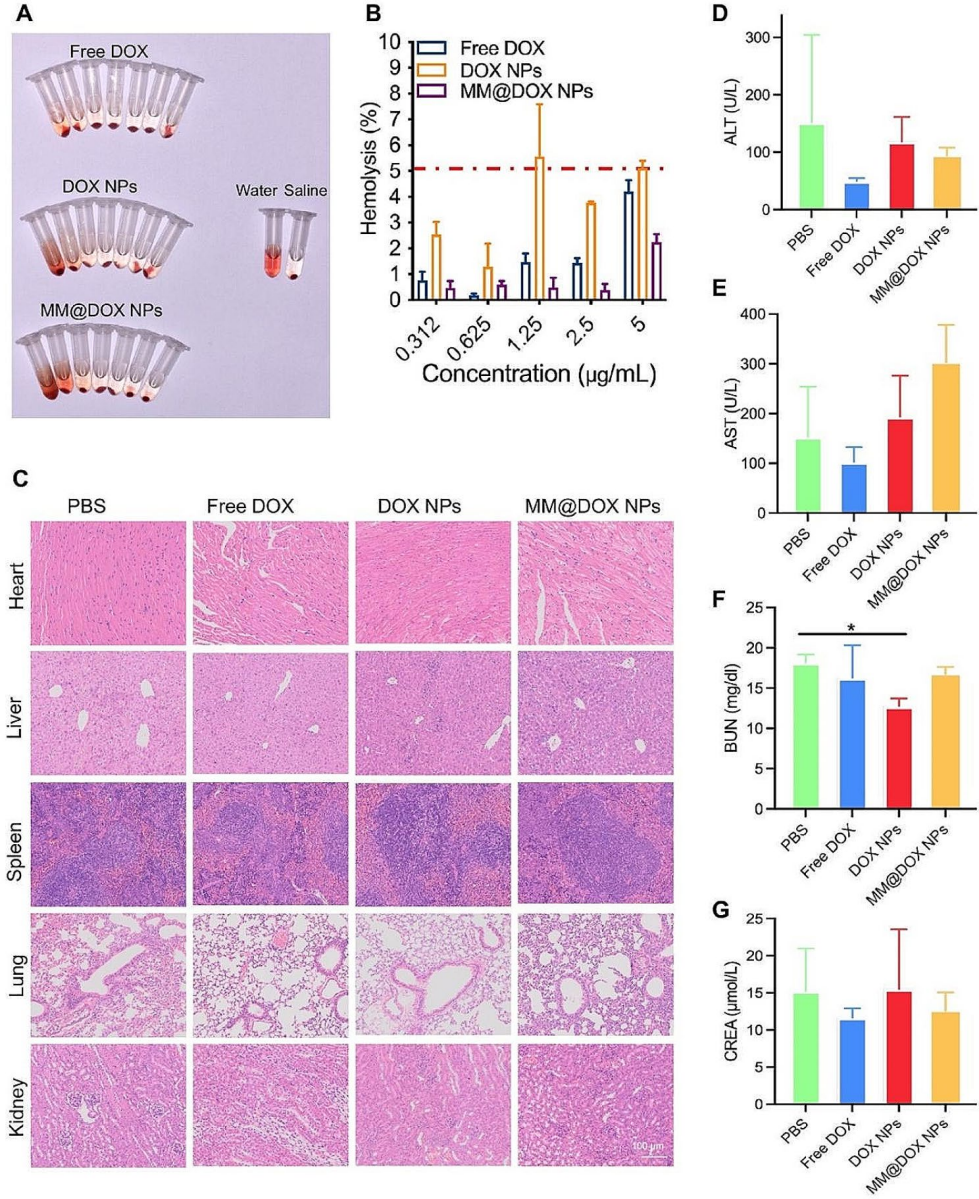
**A** Photograph of hemolysis experiments of different drugs at concentrations of 20, 10, 5, 2.5, 1.25, 0.625, 0.312 µg/mL (from left to right). **B** Calculation of hemolysis rates of free DOX, DOX NPs and MM@DOX NPs. **C** H&E staining slices of major organs including heart, liver, spleen, lung, and kidney from each group. **D, E** Main blood biochemical parameters of liver function including ALT and AST. **F, G** Main blood biochemical parameters of kidney function including BUN and CREA

Biocompatibility plays an important role in the application of drug delivery carriers in vivo [53]. The biocompatibility of biomimetic nanoparticles was evaluated through an in vitro hemolysis assay. Physiological saline was used as the negative control group, and deionized water was used as the positive control group. At a concentration of 5 µg/mL, the hemolysis rate of MM@DOX NPs remained below 3%, which was significantly lower than that of free DOX (Fig. 8A-B). A hemolysis rate below 5% is safe for drug delivery systems [54]. From our research results, biomimetic nanoparticles exhibit good biocompatibility.

Low toxicity side effects, especially on major organs, are important indicators to evaluate the safety of nanoparticles for anticancer therapy [38]. To further assess the in vivo safety, the main organs, including the heart, liver, spleen, lung and kidney, from mice treated with different strategies were further analysed (Fig. 8C). The mice treated with MM@DOX NPs showed no obvious damage to these major organs. In addition, MM@DOX NPs had no significant hepatotoxicity or nephrotoxicity in vivo. Liver function indicators such as ALT and AST, as well as kidney function markers, including CREA and BUN, were comparable to those of the PBS group (Fig. 8D-G). These results demonstrated the good biocompatibility of MM@DOX NPs.

## Conclusions

In this study, novel biomimetic macrophage membrane-coated pH-responsive nanoparticles (MM@DOX NPs) were successfully constructed, which exhibited superior OSCC inhibition in vitro and in vivo. The resulting MM@DOX NPs controlled the release of DOX in a low pH tumor microenvironment, effectively delivering drugs to the tumor site with prolonged circulation time and diminished immune clearance. Moreover, the nanoparticles exhibited stronger antitumor activity than that of free DOX and a high biosafety.

In conclusion, this study presents a biosafe, long-circulation, tumor-targeted, pH-sensitive drug delivery system for the treatment of OSCC. Through the combination of biomimetic cell membranes and responsive polymer nanoparticles, a membrane encapsulation system is rationally designed that provides a basis for the delivery of chemotherapy drugs to tumors.

### Electronic supplementary material

Below is the link to the electronic supplementary material.

Supplementary Material 1

## Data Availability

All data contained in the study are in this article
