# Supplementary material for: Macrophage membrane-camouflaged pH-sensitive nanoparticles for targeted therapy of oral squamous cell carcinoma
Source: J Nanobiotechnology. 2024 Apr 12;22:168. doi: 10.1186/s12951-024-02433-4 (PMC11015647; doi:10.1186/s12951-024-02433-4)
Supplement: Supplementary file 1 — Supplementary Material 1 [file 12951_2024_2433_MOESM1_ESM.docx]

Fig. S1. The FT-IR spectra of DOX, PMVEMA, PBA, PMVEMA-PBA, and PMVEMA-PBA-DOX.


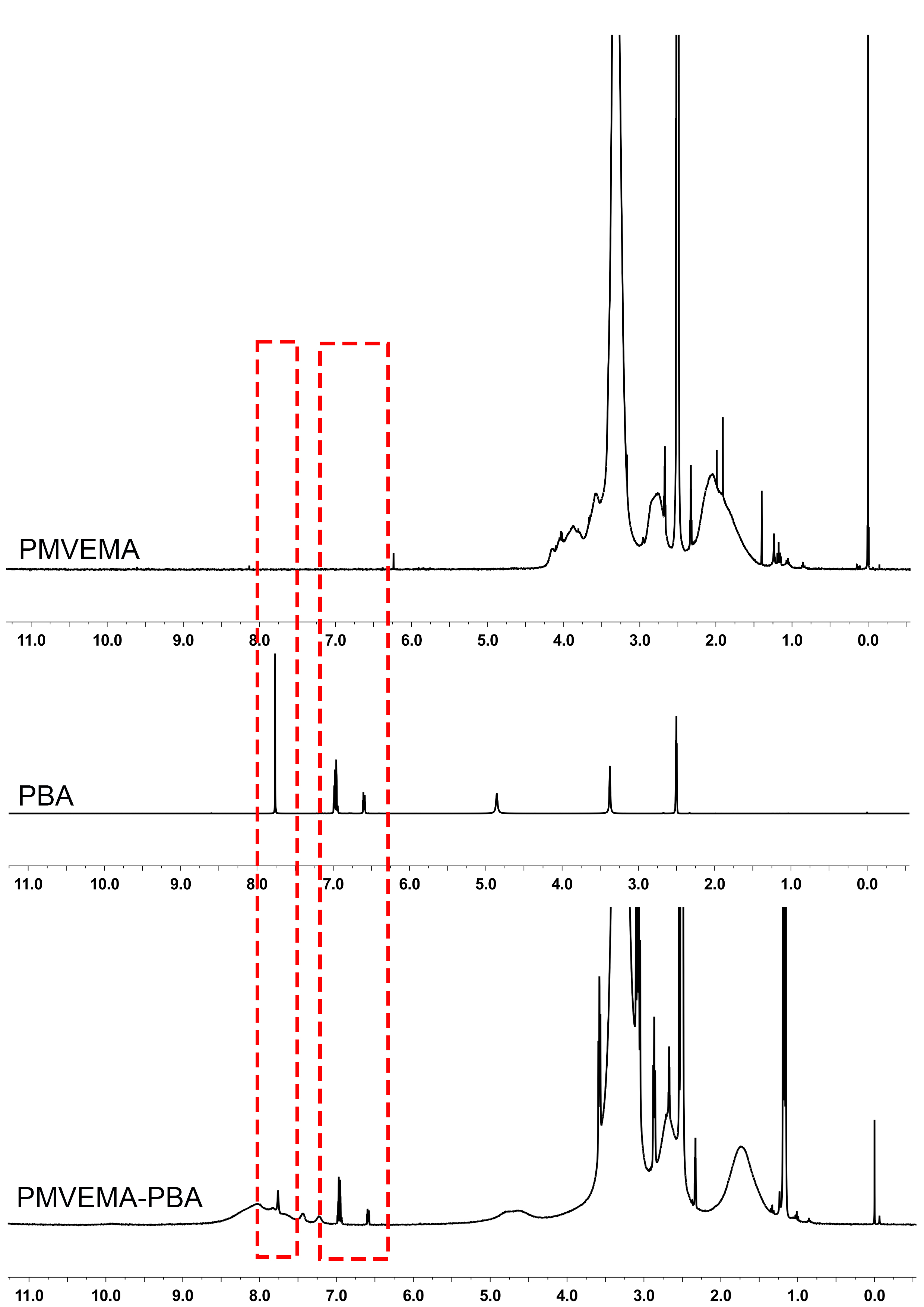


Fig. S2. The ^1^H-NMR spectra of PMVEMA, PBA and PMVEMA-PBA.


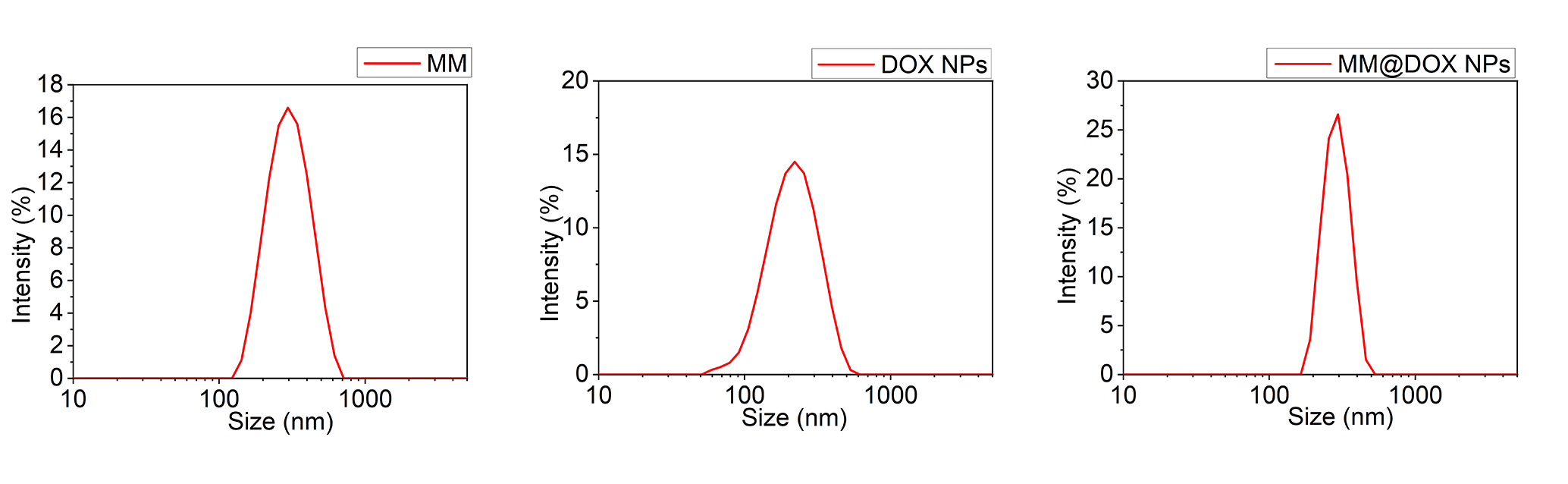


Fig. S3. MM, DOX NPs, MM@DOX NPs particle size


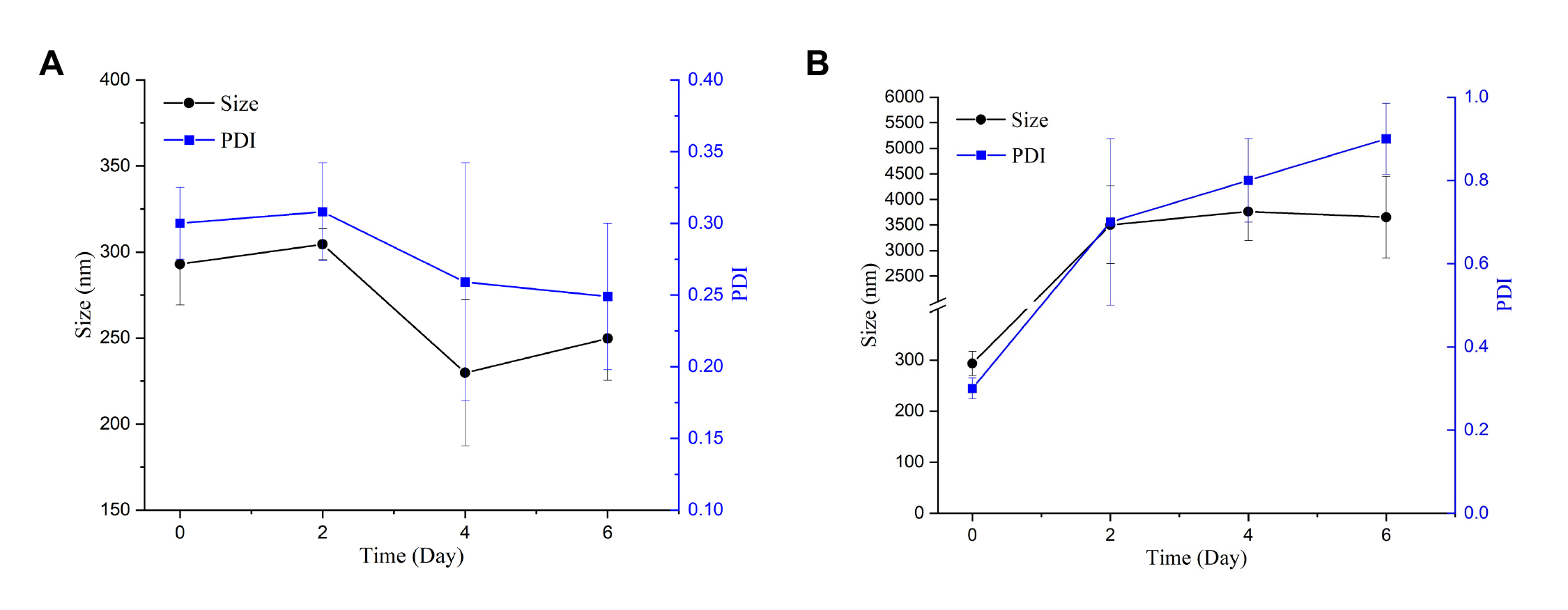


Fig. S4. **A** Stability of MM@DOX NPs in PBS under pH 7.4 detected by DLS. **B** Stability of MM@DOX NPs in PBS under pH 5.5 detected by DLS


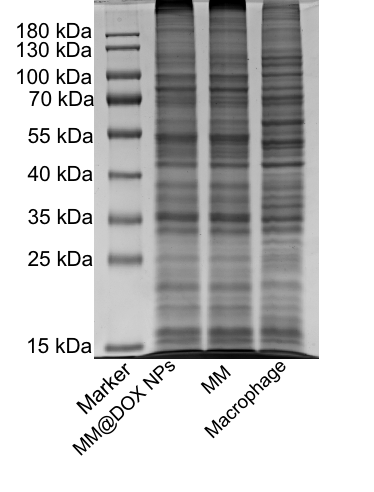


Fig. S5. Proteins in MM@DOX NPs, MM, and macrophage were characterized by polyacrylamide gel electrophoresis


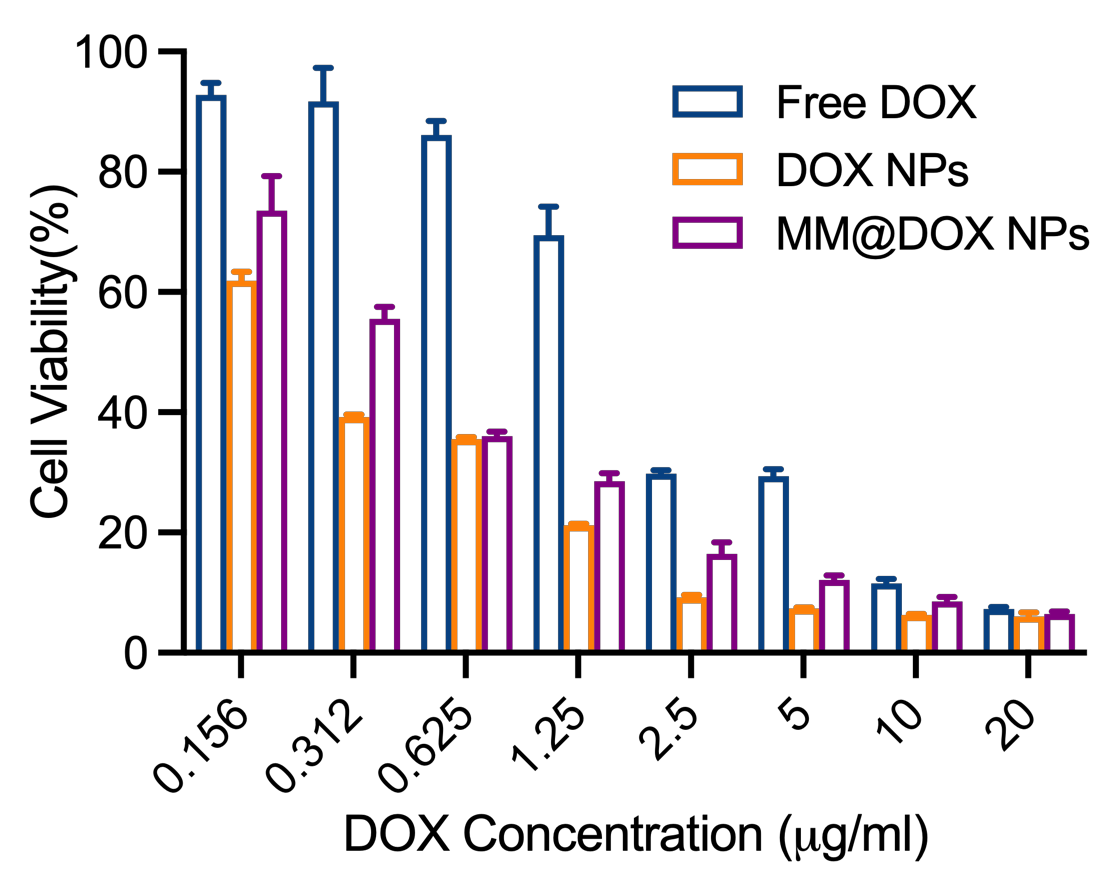


Fig. S6. HN6 cells viability after treatment with various concentration of free DOX, DOX NPs and MM@DOX NPs at pH 6.5.


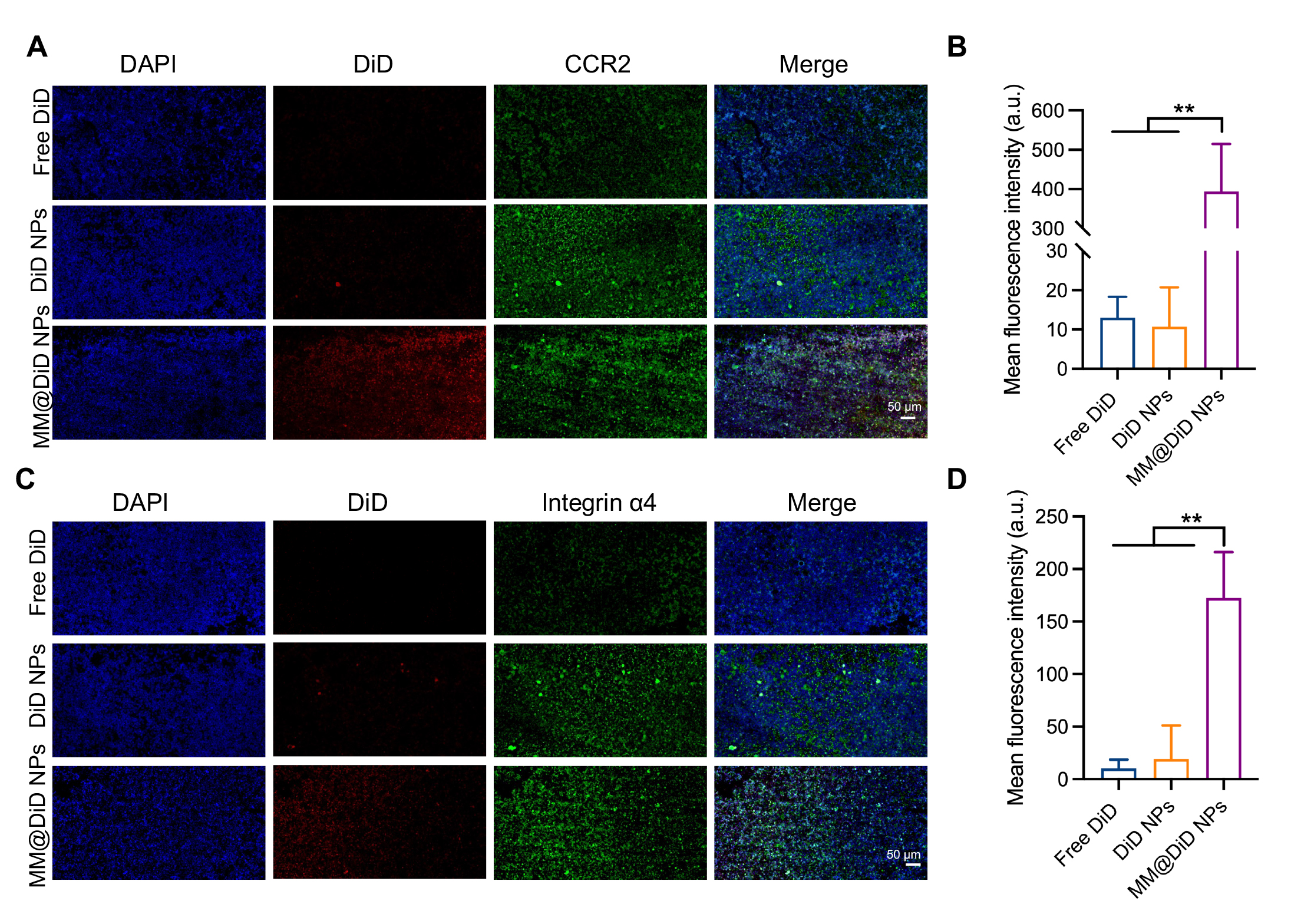


Fig. S7. **A** Representative fluorescence images of DAPI (blue), DiD (red), and CCR2 (green) in tumor of HN6 tumor-bearing mice at 24 h after intravenous injection of free DiD, DiD NPs and MM@DiD NPs, and **B** corresponding DiD fluorescence intensity (*n* = 3, mean ± SD, ** *p* < 0.05). **C** Representative fluorescence images of DAPI (blue), DiD (red), and integrin α4 (green) in tumor of HN6 tumor-bearing mice at 24 h after intravenous injection of free DiD, DiD NPs and MM@DiD NPs, and **D** corresponding DiD fluorescence intensity (*n* = 3, mean ± SD, ** *p* < 0.05).
